# Graphical data mining of cancer mechanisms with SEMA

**DOI:** 10.1093/bioinformatics/btz303

**Published:** 2019-05-09

**Authors:** Mustafa Solmaz, Adam Lane, Bilal Gonen, Ogulsheker Akmamedova, Mehmet H Gunes, Kakajan Komurov

**Affiliations:** 1 Department of Computer Science and Engineering, University of Nevada, Reno, NV, USA; 2 Division of Bone Marrow Transplantation, Cincinnati Children’s Hospital Medical Center, Cincinnati, OH, USA; 3 School of Information Technology, University of Cincinnati, Cincinnati, OH, USA; 4 Graduate Program in Mathematics, Fatih University, Istanbul, Turkey; 5 Divisions of Experimental Hematology and Cancer Biology, Human Genetics and Biomedical Informatics, Cincinnati Children’s Hospital Medical Center, Cincinnati, OH, USA; 6 Department of Pediatrics, University of Cincinnati Medical Center, Cincinnati, OH, USA

## Abstract

**Motivation:**

An important goal of cancer genomics initiatives is to provide the research community with the resources for the unbiased query of cancer mechanisms. Several excellent web platforms have been developed to enable the visual analyses of molecular alterations in cancers from these datasets. However, there are few tools to allow the researchers to mine these resources for mechanisms of cancer processes and their functional interactions in an intuitive unbiased manner.

**Results:**

To address this need, we developed SEMA, a web platform for building and testing of models of cancer mechanisms from large multidimensional cancer genomics datasets. Unlike the existing tools for the analyses and query of these resources, SEMA is explicitly designed to enable exploratory and confirmatory analyses of complex cancer mechanisms through a suite of intuitive visual and statistical functionalities. Here, we present a case study of the functional mechanisms of *TP53*-mediated tumor suppression in various cancers, using SEMA, and identify its role in the regulation of cell cycle progression, DNA repair and signal transduction in different cancers.

SEMA is a first-in-its-class web application designed to allow visual data mining and hypothesis testing from the multidimensional cancer datasets. The web application, an extensive tutorial and several video screencasts with case studies are freely available for academic use at https://sema.research.cchmc.org/.

**Availability and implementation:**

SEMA is freely available at https://sema.research.cchmc.org. The web site also contains a detailed Tutorial (also in Supplementary Information), and a link to the YouTube channel for video screencasts of analyses, including the analyses presented here. The Shiny and JavaScript source codes have been deposited to GitHub: https://github.com/msolmazm/sema.

**Supplementary information:**

Supplementary data are available at *Bioinformatics* online.

## 1 Introduction

The collection of large genomics datasets from clinical and population studies provide an unprecedented opportunity to query the molecular underpinnings of disease mechanisms. The raw and processed data from these projects have been made easily accessible to the bioinformatics community through the data portals, and tools have been developed to ease the process of acquiring and assembly of these datasets for computational analyses (Birger *et al.*, 2017; Gao *et al.*, 2013; Goldman *et al.*, 2018; Lau *et al.*, 2017; Reynolds *et al.*, 2017). Some excellent efforts have also made the data from these resources accessible to the non-computational biomedical research community (Gao *et al.*, 2013; Goldman *et al.*, 2018; McFerrin *et al.*, 2018; Newton *et al.*, 2017; Wang *et al.*, 2017; Zhou *et al.*, 2016), and still more are being developed to facilitate different aspects of data visualization, analyses and queries.

In addition to identifying the molecular aberrations in cancers, and classification of tumors based on their molecular and clinical characteristics, an important goal of cancer genomics is to identify the functional relationships of these molecular aberrations and their causal role in the clinical outcome. Cancer researchers may be interested in testing which somatic alterations lead to their phenotype of interest in cancers, or if a causal relationship they identified in an experimental model also holds in clinical samples. In fact generally, cancer biologists often deal with complex models of functional interplay between several cancer processes. However unfortunately, the existing tools are not designed to allow non-computational researchers to query the cancer genomics datasets for mechanistic models. For example, although a question such as ‘What is the rate of *TP53* mutations in cancers?’ is a question that some of these tools can be used to answer, a question such as ‘Which pathways do *TP53* mutations suppress and activate in different cancers, and how do they contribute to the poor outcome?’ is a question that cannot be answered, or even formulated, using any of the existent tools. This type of queries requires intuitive data mining and statistical modeling approaches, which are not offered in any of the existing tools for non-computational cancer researchers.

In addition to requiring models with complex multivariate structures, analyses of cancer mechanisms often can involve phenomena that are not measured by the data. For example, some of the cancer hallmarks, such as immune infiltration and angiogenesis, are important factors in cancer, and a cancer researcher may want to assess the correlation of a molecular alteration with these events. However, formulation and incorporation of such latent constructs into analyses is also an important feature that is missing from the current tools.

To address these issues in mining of cancer mechanisms from multi-omics cancer genomics data, we developed SEMA. The primary purpose of SEMA is to allow the formulation of complex multivariate hypotheses and their testing in the cancer genomics data. In SEMA, users formulate their hypotheses in the form of graphs, or networks, where nodes represent biological variables of interest (e.g. mRNA expression, clinical survival, somatic mutations), and links represent purported functional relationships between them. In this way, users can formulate multivariate hypotheses of arbitrary complexity, which allows for the testing of non-trivial relationships between a large number of variates with ease. To guide users in variable selection for exploratory analyses (e.g. ‘Which pathways do TP53 mutations activate in melanomas?’), we have compiled an extensive database of pre-computed all-against-all correlations of all the data types in TCGA (∼300 billion correlations), which the user can tap into to select the best variables to include in a model. The final user model is then analyzed for fit to the data by Structural Equation Modeling (SEM). SEM refers to an array of powerful multivariate techniques that allow for modeling of complex relationships between variables (Kline, 1998). SEM extends multivariate regression by allowing the modeling of more complex networks of relationships where variables can be response variables of some and yet predictor variables of others, which is especially useful for modeling of direct and indirect effects between variables. Functionality for interactive and comparative visualizations of the data, including as clustered heatmaps, and of the statistical results are also implemented.

## 2 Implementation

### 2.1 Interface design

**Fig. 1.**
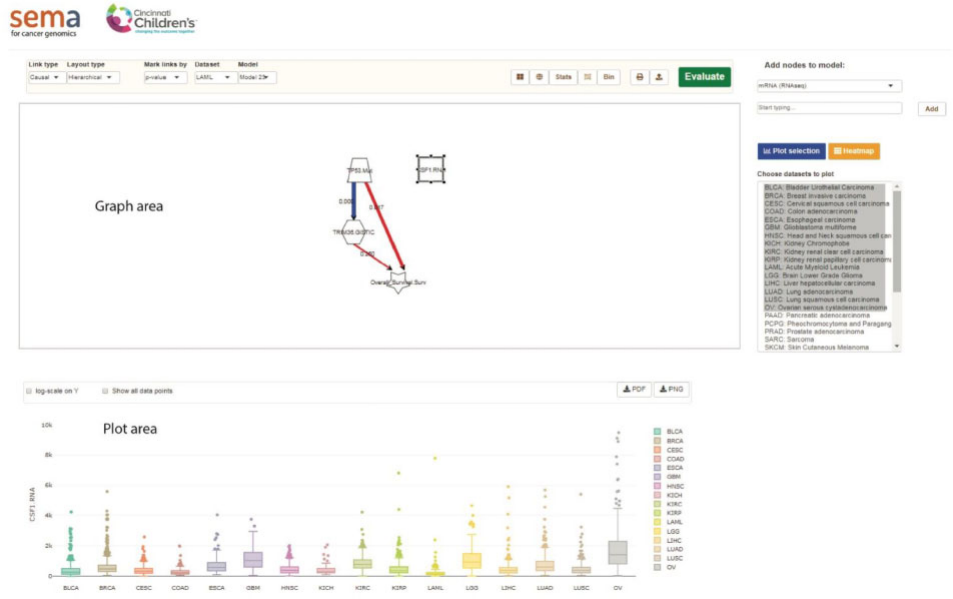
Layout of SEMA. Users build their models in the graph area (top) by adding nodes and drawing links between them. The plot area below is for visualization of the data, heatmaps and of the statistical results

The app, after launching from the home page, consists of a graph area and a plot area (Fig. 1). Users input variables into the graph, and draw links between them to formulate causal relationships to be tested. Importantly, there are no restrictions on the complexity of the graph, and users can formulate multi-component, multi-level (e.g. A causes B causes C) or even cyclic relationships. Currently, SEMA features mRNA, miRNA, protein (RPPA), somatic mutations (from whole-exome sequencing), germline variations (from whole-exome sequencing), somatic copy number variations (CNV) and clinical data from 26 cancer types (i.e. those that have >100 samples) in TCGA (Supplementary Table S1), and mRNA, somatic mutations, copy number variation and clinical data from 5 cancers in Therapeutically Applicable Research to Generate Effective Treatment (TARGET) consortium. In addition, we have included some pre-computed tumor characteristics from the PanCan immune Atlas analyses of TCGA data (Thorsson *et al.*, 2018), which includes tumor neo-antigen burden, lymphocyte infiltration, T-cell receptor richness and evenness, among many other useful metrics.

Users form hypotheses to be tested by drawing appropriate links between the variables on the graph. The variables on the graph can be visualized individually or collectively as a clustered heatmap. In addition, users can visualize pair-wise distribution of linked nodes, e.g. a box plot of the mRNA expression of a gene stratified by copy number variations in another. After the model is evaluated by SEM, users can also compare the individual statistical parameters of the links between cancer types (see Supplementary Information).

The model is statistically evaluated after the user clicks ‘Evaluate’. Statistical evaluation of the model is conducted by structural equation modeling (SEM) tool implemented in the R package *lavaan* (Rosseel, 2012). The fit statistics for the whole model can be visualized in the Model Parameters Table, while the fit statistics for the individual relationships are overlaid on the respective links on the graph and both color- and thickness-coded. Users can do comparative analyses of the fit results between cancers in the plot area (see Supplementary Information). Although there is no consensus in the field on which measures or what respective cutoffs to choose for an acceptable model fit, the general guidelines are that the comparative fit indices, e.g. Tucker-Lewis Index (TLI), should be high (e.g. TLI > 0.9–0.95), while absolute fit measures, e.g. Standardized Root Mean Square Residual (SRMR), should be low (e.g. <0.1) (Hu and Bentler, 1999). Since the chi-squared test for SEM is overly sensitive to the sample size, this test (and the associated p-value) is usually not taken into consideration when dealing with large sample sizes, and is therefore not included in the parameter table.

### 2.2 Exploratory analyses: variable selection

In the cases when *a priori* information on the gene/molecule of interest is limiting, or when a researcher simply would like to engage in an unbiased study, it is useful to do exploratory analyses. To aid in such exploratory analyses, we have compiled an extensive database of pre-computed all-against-all correlations of all the data types in TCGA (∼300 billion correlations), which the user can tap into to select the best variables to include in a model. For example, a user can identify, in an unbiased manner, the somatic mutations and copy number variations that affect cell cycle progression and overall survival in glioblastomas, include these variables in the model, and perform further analyses to obtain an optimal model to explain the regulation of cell cycle progression in these cancers (see the Tutorial in Supplementary Information, and below for a demonstration of this feature).

### 2.3 Factor analyses

In addition to the molecular and clinical variables, users can also devise latent constructs, or factors, which are not measured in the datasets, but can be deduced based on the data. For example, tumor immune infiltration, a very important phenomenon in cancers, is not reported in the data, but can be deduced based on collective mRNA expression patterns of some key marker genes. Similarly, mitotic index of tumors, differentiation (or stemness) status, etc… are some of the examples that could be depicted as factors in SEM, and analyzed with relation to molecular and clinical data. A demonstration of analyses of a complex model with factors can be found in the tutorial videos at the SEMA web site.

### 2.4 Variable manipulation

We have also implemented functionalities for custom creation of variables: binarization and grouping. It may desirable for users to categorize a numeric variable for more intuitive analyses and visualizations. For example, copy number variations (CNV) are numerically encoded data, which may be more intuitive to analyze as categorical variables (e.g. ‘Diploid’ and ‘Copy Gain’), in which case the variable could be binarized. It may also be useful to analyze some variables as a group, rather than individually. For example, one may wish to analyze the collective effect of the Ras/MAPK pathway mutations on MEK phosphorylation and survival in cancers. In this case, the individual variables (e.g. somatic mutations in *NF1*, *KRAS*, *NRAS* and *BRAF*) will be grouped under a new variable (e.g. Ras_MAPK) where the categories (e.g. ‘WT’ and ‘Mutant’) can be defined by the user (e.g. ‘WT’ if none of the individual genes are mutated, and ‘Mutant’ if any of the genes are mutated). The use of these functionalities allows for fine-grained analyses of functional associations in cancers, such as answering the question: what is the effect of *TP53*/*CDKN2A* double mutations on cell cycle progression compared to individual mutations? A video on the web site demonstrates the use of this functionality in a case study.

SEMA was developed in R Shiny and JavaScript. The code files have been deposited to GitHub (https://github.com/msolmazm/sema). For SEM analyses, the *sem* function in the R package *lavaan* is used. The graph part of SEMA uses the commercial package yFiles for HTML, which is why any potential developers wishing to improve upon or modify SEMA code will have to obtain their copy of this library to run SEMA locally.

## 3 Results and discussion

**Fig. 2.**
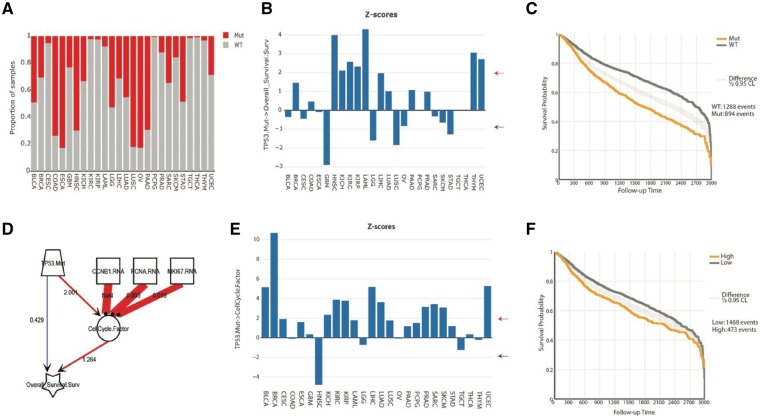
An overview of the effect of TP53 mutations on clinical outcome. (**A**) Plot of TP53 mutation rates in 26 cancers from TCGA. (**B**) Plot of the effect (z-scores, i.e. t-statistic) of TP53.Mut on overall survival in 26 adult cancers. Z-scores of absolute 1.98 correspond to the P value of 0.05 (indicated by arrows), and its direction indicates the direction of its effect. Positive effect in a survival model indicates correlation with worse outcome (i.e. positive correlation with death rates). (**C**) Kaplan–Meier plot overall survival of all cancer patients from 26 cancer types in TCGA stratified by their TP53 mutation status. (**D**) A model of the effect of TP53.Mut on overall survival and on the cell cycle, and of the cell cycle on overall survival. Causal effects are shown with directed arrows, while blunted arrows show factor definitions (here: for the cell cycle). The thickness of the links reflects the effect size (here: average across all cancers), and the color reflects the direction of the effect (red: positive, blue: negative). The numbers overlaid on the links are the z-scores (t-statistic) from SEM analyses (averaged across all cancers). (**E**) Z-scores for the effect of TP53.Mut on CellCycle.Factor in 26 adult cancers. (**F**) Kaplan–Meier curve for overall survival of patients in 26 cancers stratified by high or low values for CellCycle.Factor. All the figure panels were produced in SEMA

As a show-case of integrated pan-cancer analyses in SEMA, we have analyzed the effect of *TP53* mutations (*TP53.Mut*) on clinical outcome, and the potential mechanisms of its action across human cancers. Mutations in *TP53* are very common in human cancers, with the highest rates observed in Esophageal (ESCA), lung squamous (LUSC) and ovarian (OV) cancers (Fig. 2A). As expected, *TP53.Mut* also correlated with poor outcome in several cancers (HNSC, KICH, KIRC, KIRP, LAML, LIHC, THYM and UCEC), though it correlated with better outcome in glioblastomas (GBM) and low-grade gliomas (LGG) (Fig. 2B–C). A quick analysis of global correlates of *TP53.Mut* in GBM and LGG reveals that the correlation of *TP53.Mut* with better outcome in GBM and LGG is largely due to the confounding effect of *IDH1* mutations in these cancers, which significantly co-occurs with *TP53.Mut* (Supplementary Fig. S1A). Controlling for *IDH1* mutations in this model shows that *TP53.Mut* does not have a significant correlation with survival in these cancers (Supplementary Fig. S1B).

The results in Supplementary Figure S1 showcase the effect that confounding factors can have on the interpretation of a hypothesis (model) in the large genomic datasets. As with most statistical modeling tools, users should be careful to avoid some common pitfalls associated with the interpretation of such correlations. SEM is essentially a hypothesis testing tool (i.e. it is for confirmatory analyses), and is sensitive to the misspecification of the proposed associations between variables. Thus, the goodness-of-fit indices or the strength of associations of individual links in a model only reflect how well the model fits the data, and do not necessarily imply that the hypothesis is biologically correct. In the analysis of the effects of *TP53* mutations above, this is illustrated by the frequent co-occurrence, or mutual exclusivity, of the oncogenic events in cancers, which may have confounding effects on their correlations with other molecular and clinical parameters (e.g. co-occurrence of *TP53.Mut* with IDH1 mutations in GBM and LGG, Supplementary Fig. S1). Therefore, the researchers are strongly encouraged to try different versions of their hypotheses by employing the exploratory analyses like described above and below, with the inclusion and exclusion of additional suspected players, and testing the model fit across different datasets, to ensure robustness of the results and prevent overfitting.

Next, we asked how *TP53.Mut* leads to poor outcome in HNSC, KICH, KIRC, KIRP, LAML, LIHC, THYM and UCEC. First, we tested the best-characterized function of p53: the regulation of cell cycle. To model cell cycle regulation, we designed a latent factor (*CellCycle.Factor*) that was defined by the mRNA expression of CCNB1, PCNA and MKI67, three well-established markers of active proliferation. As expected, *TP53.Mut* had a positive effect on *CellCycle.Factor* in most cancers (Fig. 2D–E); and *CellCycle.Factor* in turn was also associated with worse survival in many cancers (Fig. 2F). Importantly, including *CellCycle.Factor* in the model reduced the direct effect of *TP53.Mut* on the clinical outcome in KICH, KIRP and KIRC and LIHC (Supplementary Fig. S2), indicating that the main effect of *TP53.Mut* on clinical outcome in these cancers was through promoting the cell cycle progression.

Since the inclusion of *CellCycle.Factor* in other cancers where *TP53.Mut* has a significant effect on clinical outcome (i.e. UCEC, THYM, LAML and HNSC) did not lessen the direct effect of *TP53.Mut* on the clinical outcome, the role of *TP53.Mut* in these cancers might be due to its role in other pro-tumorigenic processes. To find out the genes whose regulation mediates the effect of *TP53.Mut* on the outcome, we performed an exploratory analysis of global correlates of *TP53.Mut* in each cancer. Interestingly, in UCEC, one of the highest correlates with both *TP53.Mut* and overall survival was the mRNA (from RNAseq) and protein (from RPPA) expression of ESR1, the estrogen receptor. *TP53.Mut* was significantly negatively associated with ESR1 (Supplementary Fig. S3A–B), suggesting that *TP53.Mut* may be mutually exclusive with the hormone receptor status in the endometrial carcinomas. Importantly, including ESR1 mRNA expression in the model completely abolished the direct effect of *TP53.Mut* on the overall survival (Supplementary Fig. S3C), suggesting that the effect of *TP53.Mut* on the overall survival in UCEC was due to its mutual exclusivity with ESR1 expression, which predicts significantly better survival in UCEC (Supplementary Fig. S3C–D).

**Fig. 3.**
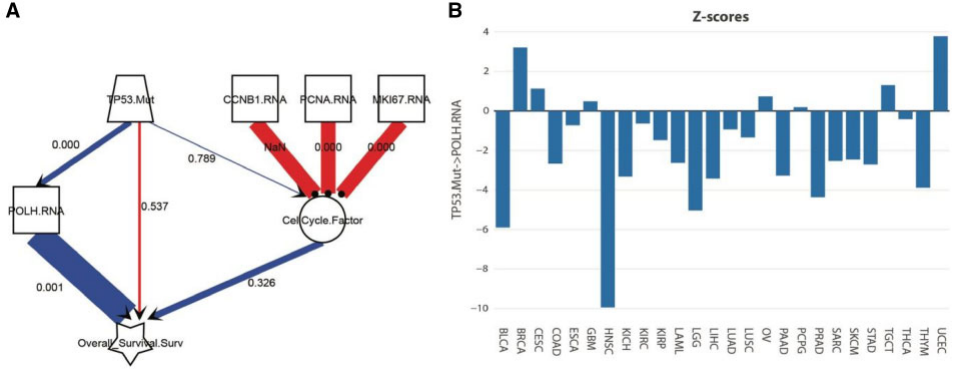
Regulation of POLH expression by TP53.Mut contributes to poor outcome in thymomas (THYM). (**A**) Model showing the effect of TP53.Mut on overall survival with the inclusion of a mediating effect of POLH mRNA expression. The numbers on the links show P-values for the effect in THYM. (**B**) Z-scores for the effect of TP53.Mut on POLH mRNA expression in 26 adult cancers. All the figure panels were produced in SEMA

A similar analysis in THYM revealed POLH mRNA expression as the highest correlate of *TP53.Mut* (Fig. 3A). POLH codes for DNA polymerase η (eta), which is involved in DNA repair and hypermutation during immunoglobulin class switch recombination, and is a known target of p53 (Fischer, 2017). Accordingly, it is frequently suppressed in *TP53*-mutant cancers (Fig. 3B). The inclusion of POLH in the model also abrogated the direct effect of *TP53.Mut* on survival (Fig. 3A), likewise suggesting that POLH is one of the most important targets of *TP53.Mut* in THYM, and shows the indispensable tumor suppressor role of POLH in thymomas.

**Fig. 4.**
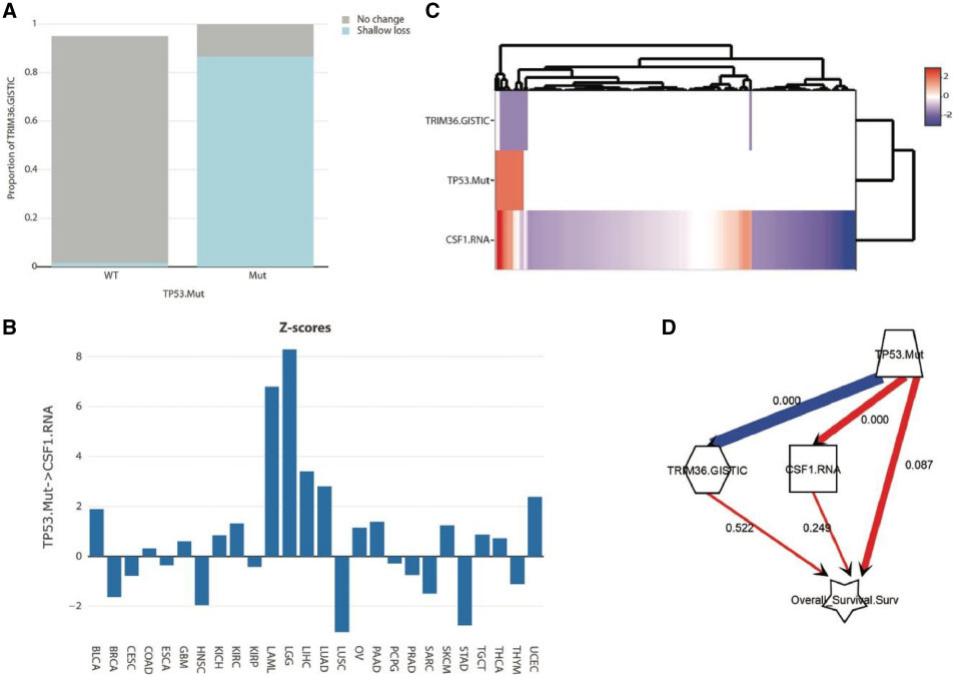
Decoding the effect of TP53.Mut in LAML. (**A**) Stacked barplot showing the co-occurrence of TP53.Mut with TRIM36 loss (i.e. chromosome 5 monosomy/shallow loss). (**B**) The z-scores of the effect of TP53.Mut on CSF1 mRNA expression in 26 adult cancers. (**C**) Heatmap of the indicated variates in LAML patients. The values in the rows were scaled. Somatic mutations are white when wild-type, and light red when mutated. For copy number variations, white indicates no change, pink: gain, red: amplification, light blue: shallow loss and deep blue: deep loss. RNA expressions are z-transformed according to the color key. (**D**) Model of the effect of TP53.Mut on overall survival after including the mediating effects of TRIM36.GISTIC and CSF1.RNA. The overlaid numbers are the P-values for LAML. All the figure panels were produced in SEMA

In Acute Myeloid Leukemia (LAML), there were no significant correlates among frequent somatic mutations. Then, we looked at the copy number variations that correlated with both *TP53.Mut* and overall survival, and identified *TRIM36* as the top candidate, along many other genes. *TRIM36* is on chromosome 5, which is frequently lost (monosomy 5) in myeloid neoplasms, including AML (Le Beau, 1992). Importantly, *TP53.Mut* significantly co-occurs with the loss of *TRIM36* in LAML (Fig. 4A). Controlling for *TRIM36* loss reduces the effect of *TP53.Mut* on overall survival in LAML, although not completely (p-value of effect of *TP53.Mut* on overall survival after controlling for *TRIM36* loss = 0.017), suggesting that *TP53.Mut* has significant effect on overall survival independent of its co-occurrence with chromosome 5 loss. To identify this effect, we examined the mRNA expression correlates of *TP53.Mut* and overall survival. To eliminate any confounding effect of *TRIM36* copy number changes, we also included *TRIM36* copy number variations in this analysis. We looked for gene mRNAs that positively correlated with both *TP53.Mut* and overall survival, but did not significantly correlate with *TRIM36* loss. The top 2 genes identified in this manner were FHL2 and CSF1. An analysis of the effect of *TP53.Mut* on FHL2 expression across cancers showed that it is not frequently associated with *TP53.Mut* (not shown), suggesting that it might not be a direct *TP53.Mut* target. However, CSF1 expression was significantly upregulated in *TP53.Mut*-dependent manner in 6 cancers (Fig. 4B), suggesting that CSF1 may be a direct target of *TP53.Mut* in many cancers, including LAML (Fig. 4C), as also reported previously (Fischer, 2017). Importantly, including CSF1 mRNA expression in the model significantly reduced the effect of *TP53.Mut* on overall survival (Fig. 4D), suggesting that the regulation of CSF1 expression by *TP53.Mut* contributes to its pro-tumorigenic effect in LAML. It is worth noting that CSF1-CSF1R signaling has a well-established oncogenic role in LAML (Edwards *et al.*, 2019), and our analysis here links somatic mutations in *TP53* to the activation of this pathway in several cancers. All of the analyses presented here were performed using only SEMA functionalities.

## 4 Conclusion

SEMA is a web application to test simple and complex hypotheses in the form of graphs using the powerful SEM approach. By allowing the inclusion of different data types (e.g. protein, RNA, mutations, clinical survival) in a single path model, SEMA provides a platform for integrative analyses of multidimensional data. Unlike other platforms, such as cBioPortal and UCSC Xena, where the focus is on enabling the analyses and visualizations of individual molecular aberrations in cancers, SEMA is focused on enabling the mining of complex relationships between molecular aberrations. Users can engage in confirmatory analyses, or perform exploratory analyses, through the use of a suite of intuitive visual and statistical tools. SEMA is the only available tool that can be used for this type of analyses. Although it currently only features TCGA and TARGET datasets, we will expand the database to include more multi-dimensional datasets in the near future. As with any computational tool, the broader and deeper functionalities in SEMA will require a learning curve on the part of the users, especially those who are new to statistical modeling and related concepts. To facilitate the familiarization of researchers with the use of functionalities in SEMA, the tutorial on the web (and Supplementary Information) demonstrates steps for hypothesis building and testing using basic path models and factor analyses in SEMA. In addition, we have built a YouTube channel for SEMA, featuring screencasts of various analyses in SEMA, including for the analyses presented above. The YouTube channel will be continuously updated with new videos of analyses, demonstrations of newly introduced functionalities, and the analyses submitted by the user community.

## 5 Requirements

*Web browser compatibility*: SEMA has been tested to run on Chrome, Firefox and Safari. SEMA is not compatible with the Internet Explorer and Edge.

*Programming language:* SEMA was written in Shiny and JavaScript.

*License:* GPL 3.0.

## Funding

This work was supported by the Center for Pediatric Genomics (CpG) grant from the Cincinnati Children’s Hospital Medical Center, and partially by NCI (CA193549) and National Science Foundation (EPS-IIA-1301726).

*Conflict of Interest*: none declared.

## Supplementary Material

btz303_Supplementary_Materials
