# Supplementary material for: Graphical data mining of cancer mechanisms with SEMA
Source: Bioinformatics. 2019 May 9;35(21):4413–8. doi: 10.1093/bioinformatics/btz303 (PMC6821276; doi:10.1093/bioinformatics/btz303)
Supplement: btz303_Supplementary_Materials [file btz303_supplementary_materials.pdf]

Supplementary Materials

Contents:

- **The SEMA Tutorial**
- **Supplementary Figures**

Supplementary Figure legends

Supp. Fig. 1. Confounding effect of *IDH1* mutations on the effect of *TP53.Mut* on overall survival in gliomas. A) Plot showing co-occurrence of *IDH1.Mut* and *TP53.Mut* in LGG and GBM. B) Models with overlaid respective *P*-values of *TP53.Mut* effect on overall survival with the mediating effect of *IDH1.Mut*. Compare with Fig.2B.

Supp. Fig. 2. The effect of *TP53.Mut* on overall survival that is not explained by *CellCycle.Factor* in 26 adult cancers. Compared with Fig.2B. Those cancers where the direct effect of *TP53.Mut* that is independent of *CellCycle.Factor* is significantly reduced are marked with an arrow.

Supp. Fig. 3. The confounding effect of estrogen receptor status on *TP53.Mut* effect on survival in UCEC. A) Heatmap of the indicated variates in UCEC patient samples. Note the co-occurrence of ESR1-low population with *TP53.Mut*. B) mRNA expression of ESR1 stratified by *TP53.Mut* status. C) Model of *TP53.Mut* effect on survival mediated by *CellCycle.Factor* and ESR1.RNA in UCEC. Overlaid numbers are the respective z-scores for each link. D) Kaplan-meier curve of overall survival in UCEC stratified by low, medium or high expression of ESR1 (0-33, 33-66 and 66-100 percentiles of ESR1 expression, respectively).

SEMA Tutorial

Table of Contents

What is SEMA? 1

How do we start? 1

Visualize data as heatmaps 5

Exploratory analyses 5

Factor analyses 7

What is SEMA?

SEMA is a web platform for mining of functional mechanistic relationships from the large cancer genomics datasets. Currently, data from The Cancer Genome Atlas (**TCGA**: 26 adult cancer types) and Therapeutically Applicable Research to Generate Effective Treatment (**TARGET**: 5 pediatric cancer types) initiatives are available for analyses in SEMA. Unlike other platforms, such as cBioPortal and UCSC Xena, where the focus is on enabling the analyses and visualizations of individual molecular aberrations in cancers, SEMA is focused on enabling the mining of complex relationships between molecular aberrations. Users can engage in confirmatory analyses, with questions such as "Do mutations in *TP53* affect cell cycle progression or immune infiltration, and which of these functions has a stronger effect on outcome?", or perform exploratory analyses, e.g. "Which somatic alterations (mutations and copy number variations) activate the cell cycle, suppress immune cell infiltration and correlate with poor outcome in cancers?"

How do we start?

In SEMA, users build hypotheses, or models in the graph window (see below in Figure 1).

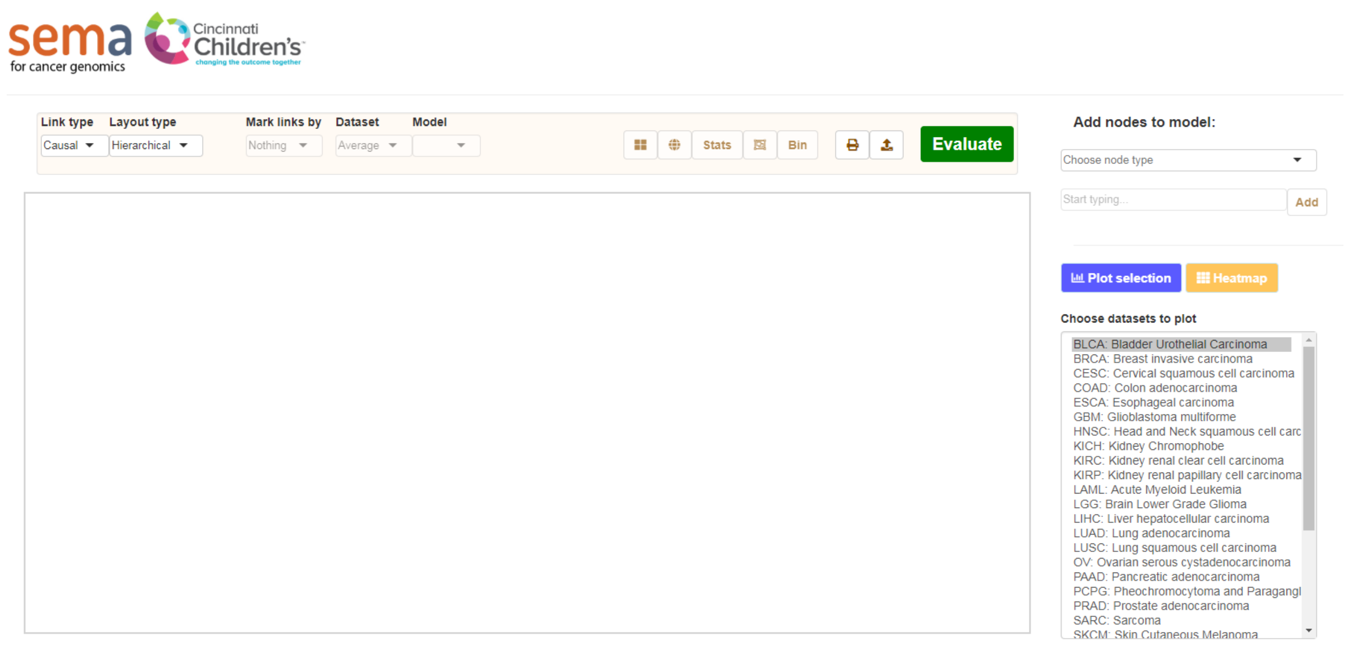

Figure 1. The graph area in SEMA. Users start by adding variables (mRNA/miRNA expression, somatic or germline mutations, somatic copy number changes, clinical variables, etc…) to the graph (model) at the top right corner (under "Add nodes to model"). The functions of the buttons and drop-down boxes above the graph are described in Table 1.

Users start by adding variables of interest to the graph to make a model (hypothesis) to be tested. SEMA features 10 types of molecular and clinical variables for analyses (Table 2).

Input name/type	Description
Link type	Select link type to draw between two nodes, this is either "factor" or "causal" (default). Causal links define potential causal relationships to be tested, while factor links are for defining factors.
Layout type	Choose the type of layout for the model graph. The graph will update its layout when you change the layout type
Mark links by	After the model is evaluated by SEM, this option will be activated. You can overlay the p-values, coefficients or z-scores on top of the links to visualize their statistics calculated by SEM.
Dataset	This is to choose a dataset for which the statistics on the links will be visualized on the graph.
Model	Each evaluated model will be saved here (in order). You can retrieve previous evaluated models by selecting them here.
View covariance matrix button	This will show the correlation heatmap of all the variables in the model. This is useful to guide what links to include in the model
View global correlations of selected variables button	This will show a table of correlations of selected variables in the model with all other variables of selected type in selected dataset. This is a useful functionality to choose variables to include in the model
Stats button	This will show the fit statistics of each evaluated model. Several fit measures are provided, and the user can compare different versions of models
Combine variables button	This will allow the user to combine selected variables in the model into one based on some logic. Refer to the tutorial (doc and video) for illustrations of this function.
Bin button	This is to binarize (categorize) a numeric variable. This can be useful when the user wants to categorize, e.g. mRNA expression, into binary categories, e.g. "Low" and "High".
Print	Print the model
Export button	User can export the model to an image format, or download the raw data of variables in the model.

Table 1. Description of functions for the model graph.

For this tutorial, choose "Somatic mutations" in the "node type", type "TP53" in the text field below, and choose "TP53" from the suggestions in the drop-down menu, and click "Add" button. Now, the "TP53.Mut" variable, depicting somatic *TP53* mutations in cancers, has been added to the model.

Variable type	Explanation
mRNA (RNAseq)	Gene-level mRNA expression data from RNAseq [1]
miRNA (miRNAseq)	miRNA expression data from miRNAseq [1]
Protein (RPPA)	Protein measurements from the reverse-phase protein array data [1]
Somatic mutation (WXS)	Somatic mutations from whole-exome sequencing data [1]
Germline variation (WXS)	Germline variations from whole-exome sequencing data [2]
Copy number variation (SNP6)	Copy number variations from the SNP6 array data [1]
Copy number variation (GISTIC)	Copy number variation calls (one of "Deep loss", "Shallow loss", "No change", "Gain" or "Amp") by GISTIC from SNP6 array data [3]
Tumor features (PanCan iAtlas)	Various tumor featuress from the PanCan iAtlas dataset [4]
Clinical	Clinical data, including survival data [5]
Factor	Custom creation of a latent variable

[1] From GDAC Firehose
[2] From Huang et al Cell (2018)
[3] From Taylor et al Cancer Cell (2018)
[4] From Thorsson et al Immunity (2018)
[5] Liu et al Cell (2018)

Table 2. Variable types featured in SEMA, their explanation and their source. The sources displayed are for TCGA data. All of the TARGET data was downloaded from cBioPortal.

To visualize the distribution of *TP53* mutations across tumor types, click on the node in the graph, and press "Plot selection" button on the right. A stacked bar plot will appear showing the distribution of samples with wild-type (WT) or mutant (Mut) samples in the selected cancers. The type of plot will change depending on the variable type: it will be boxplots in the case of continuous type data (e.g. mRNA, miRNA or Protein expression data), and Kaplan-meier plot in the case of survival data (see below). The cancer types to be displayed is selected on the list below the "Plot selection" button. Select all of the cancers on the list to visualize the distribution of *TP53* mutations across different adult and pediatric cancers. You can change the Y-axis options in the plot by selecting "Proportion" instead of "Total numbers" to view the rate of *TP53* mutations, rather than absolute numbers (Figure 2).

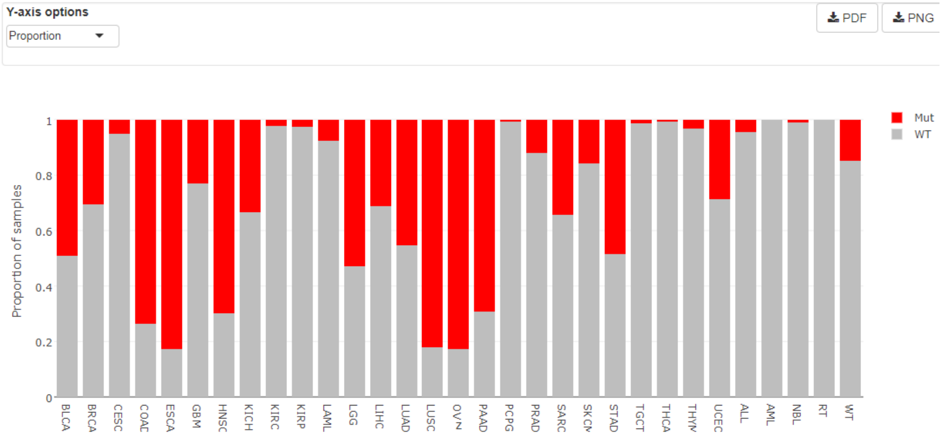

Figure 3. Visualizing the distribution of TP53 *mutations across cancers.*

A hypothesis consists of variables and their purported causal relationships, which then can be tested for fit to the data. Let's first test if *TP53* mutations correlate with poor prognosis in cancers. Select "Clinical" for the node type, and select "Overall_Survival" as the clinical variable, and add it to the model. You can visualize the overall survival of patients as a function of time in each cancer by clicking on the

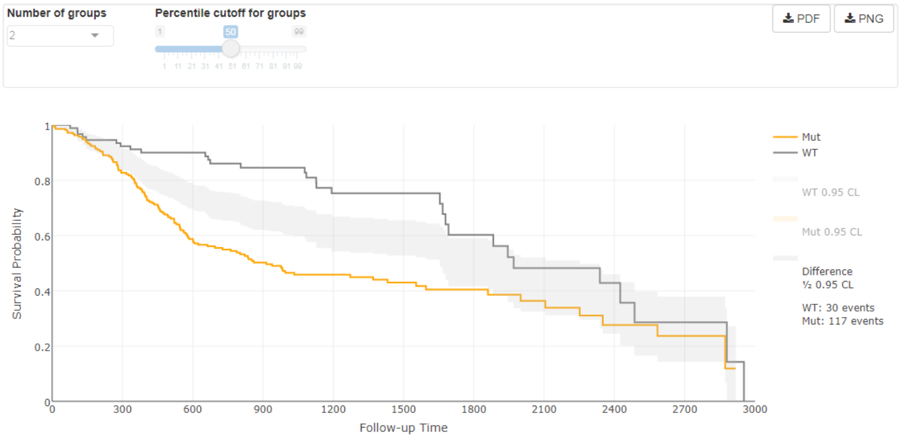

Figure 2. Kaplan-meier plot of overall survival stratified by TP53 mutations in head-and-neck cancers (HNSC) from TCGA.

Overall_Survival.Surv node and clicking “Plot selection”, which will show the Kaplan-meier plots of patient survival in the selected cancers. To make the hypothesis stated above, draw a link from TP53.Mut node to the Overall_Survival.Surv node by clicking on TP53.Mut and dragging a line to Overall_Survival.Surv. Now we have a testable model (hypothesis) in the graph that can be tested. If you now click on the link between TP53.Mut and Overall_Survival.Surv nodes and click “Plot selection”, a Kaplan-meier plot will show overall patient survival stratified by TP53.Mut in the selected cancers (Figure 3). The type of plot will change depending on the node types on the link: it will be a scatter plot if the types of both nodes on the link are continuous, or boxplot groups if one is continuous (mRNA expression) and the other is a categorical variable (e.g. somatic mutations).

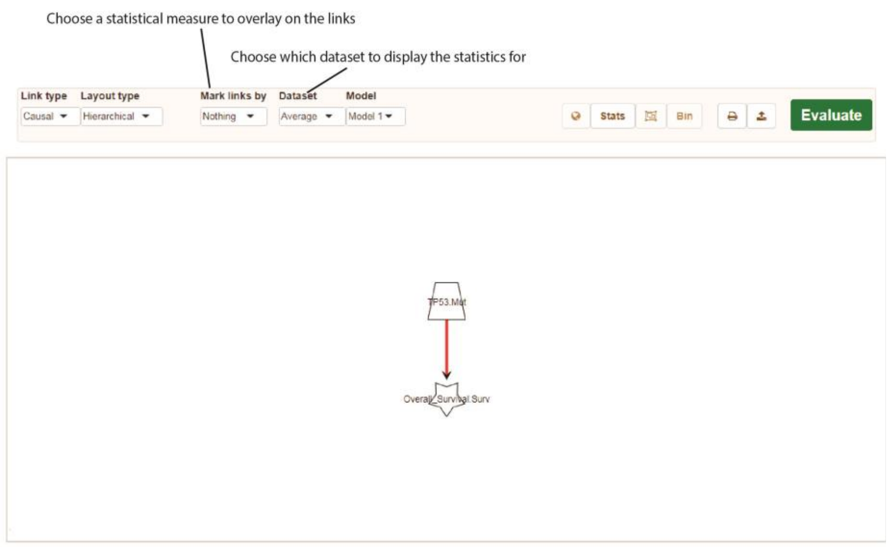

Figure 4. After evaluation of the model, the links in the model are color- and thickness-coded based on the calculated effect size (β coefficient). Red indicates a positive association, blue indicates a negative association. The thickness reflects the effect size. The z-score (β/s.e.) or p-values for each link can be optionally displayed by choosing the respective options in the “Mark links by” dropdown menu. By default, the average of these selected values across all datasets (TCGA and TARGET) will be shown. However, the user can choose individual cancers to display the statistics just for specific datasets.

Formal evaluation of the model for statistical fit is done by clicking “Evaluate” button on top of the graph. After evaluation of the model, the color and thickness of the links will change based on the statistics of each link, and different fit statistics can be displayed on the links (Figure 4). Users can also compare the fit measures between cancers by selecting the link, clicking on “Plot selection”, and choose a fit measure from the drop-down menu “Plot for the link:” which now appeared on top of the plot area. Choose “P-values (-log10)” to compare the p-values of TP53.Mut → Survival correlation between cancers, and select all cancer types in the “Choose datasets to plot” list to compare how TP53 mutations affect survival in different cancers (Figure 5).

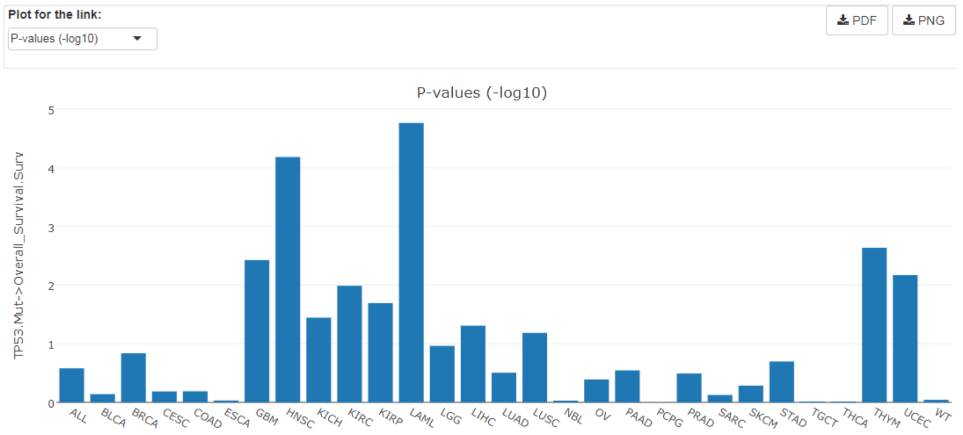

Figure 5. Comparative analysis of correlation of TP53 mutations with survival across cancers.

SEMA allows for testing of models of arbitrary complexity in a multivariate manner. Next, add a node for CDKN2A copy number loss, another frequent event in cancers. Select “Copy Number Variation (GISTIC)” as the node type (see Table 1 for explanation of node types), and type CDKN2A in the text field, select CDKN2A, and click “Add”. Draw a link between the newly

created CDKN2A.GISTIC node and Overall_Survival.Surv. Now, we have a multivariate hypothesis asking the *relative* effect of TP53 mutations and CDKN2A loss on overall survival in the clinic. Click “Evaluate” and analyze the fit statistics of each link like above. Note that the link statistics now reflect the effect of each variable (TP53.Mut and CDKN2A.GISTIC) having controlled for the other; thus, it reflects their partial effect on survival.

You can complicate the model by adding PCNA mRNA expression, a marker of cell cycle progression, and ask how TP53 mutations and CDKN2A loss affect cell cycle progression across different cancers, and if PCNA mRNA expression in turn affects overall survival.

Visualize data as heatmaps

Once there are at least two variables in the model, the user can visualize their overall all-against-all correlation matrix in each cancer by clicking on the button, which will open a dialog window with a heatmap matrix. The user can also visualize the heatmap of data values in the model across the patient samples in a given dataset by clicking on the orange “Heatmap” button next to the “Plot selection” button on the right of the graph area. These heatmaps are interactive, allowing users to zoom in and out of different regions of the heatmap for finer analysis of the data. Somatic mutations are depicted as white for WT and red for mutant categories, while GISTIC copy number changes are coded as deep blue for deep loss, light blue for shallow loss, white for no loss, pink for gain and red for amplifications.

Exploratory analyses

In addition to confirmatory analyses, like the ones described above, users can engage in exploratory analyses. This is especially useful when one does not have prior knowledge about his molecule of interest. SEMA allows for exploratory analyses by providing all pair-wise correlations of all the variables in the datasets with the variable(s) of interest in the model. The user can then make informed decisions about which variables to include in his model for further analyses.

To illustrate this, click on TP53.Mut node in your model, and press on the button, which will open up a table showing all the genes whose RNA expression correlates with TP53 mutations in BLCA (bladder cancer from TCGA). Be patient while the table is populated with the data. You can choose a different variable to compare TP53 mutations against, and view correlations in a different dataset. The table columns are sortable, so you can sort the values based on positive or negative correlations. The selected variables can be imported to the model by clicking on the button at the bottom of the table.

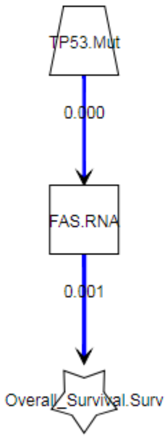

Figure 6.

Importantly, you can compare two variables at the same for an even more granular approach. Now select TP53.Mut and Overall_Survival.Surv nodes at the same time, and click on the button again. Now you have two columns of correlations, one for each variable of interest. It is possible now to select variables based on the pattern of their correlation with TP53 mutations and with the clinical outcome. Note that the range of values in the TP53.Mut column is different from that in the Overall_Survival column, as the latter shows

z-scores from a Cox regression analysis, while the correlations for all the other variables reflect the Pearson's *r* values (ranges from -1 to 1). For this illustration, let's choose genes whose RNA expression negatively correlates with TP53 mutations and that predict better survival (i.e. negative Cox z-score) in malignant melanomas (choose "RNA" under data type, and "SKCM" for dataset). In other words, we are looking for genes that are suppressed when TP53 is mutated, but would predict better survival when they are overexpressed in melanomas. To do this, first filter the TP53.Mut column at the bottom to include only genes that have < -0.15 r correlation values. Now, sort the Overall_Survival column from low to high. The top gene is FAS, the receptor for the death receptor ligand FasL, which mediates immune-mediated apoptosis in cancer cells, and is a well-known target of the p53 tumor suppressor. Select this gene and import into the model.

Remove all the nodes except TP53.Mut, Overall_Survival and FAS.RNA nodes. Now draw a causal link from TP53.Mut to FAS.RNA, and from RAS.RNA to Overall_Survival, and click on "Evaluate". Select "SKCM" in the Dataset menu above, and choose "p-value" to mark the links. The result should be like shown in Figure 6. You can analyze how these values hold across cancers like described above; e.g. compare z-scores of TP53.Mut→FAS.RNA correlations across cancers and see the consistency, indicating that the FAS gene may be an important target of the p53 tumor suppressor in cancers.

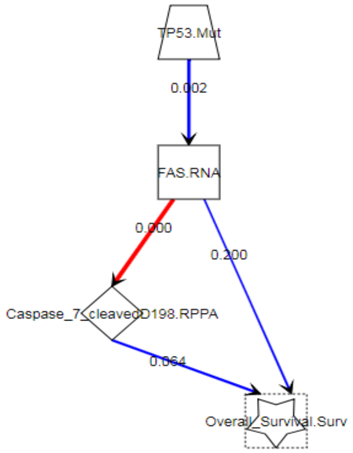

Figure 7

You can continue this analysis by now exploring all the global correlates of FAS.RNA in SKCM by following the steps above. If you specifically look at the global protein-level correlates (from RPPA) of FAS.RNA in SKCM, you will see cleaved Caspase 7 as the top correlate, which is a marker of active immune-mediated tumor lysis. To test the role of cleaved Caspase 7 in FAS.RNA-mediated favorable prognosis, add this variable to the model, and draw a link to it from FAS.RNA, and from it to Overall_Survival. Click "Evaluate". The results should be like shown in Figure 7. Analyze the p-values of correlations, and you will notice strong ties from FAS.RNA to Caspase_7_cleaved node, and from that to Overall_Survival. However, the p-value of the link from FAS.RNA to Overall_Survival has now lost its significance as its p-value increased to >0.2. This indicates that Caspase_7_cleaved node ***mediates*** most of the effect of FAS.RNA on Overall_Survival, thereby suggesting that the tumor suppressor role of FAS expression in SKCM is primarily through inducing immune-mediated tumor lysis. All in all, in this exploratory analysis, we demonstrated that **increased FAS mRNA levels in tumors lead to the susceptibility to anti-tumor immune attack, tumor lysis and better survival in malignant melanoma patients. However, mutations in TP53 disrupt the expression of FAS mRNA, and potentiate poor prognosis**.

Factor analyses

Multivariate complex models in SEMA are evaluated at the back-end by [Structural Equation Modeling (SEM)] implemented in the R package *lavaan*. In addition to its ability to model complex structures of relationships between multiple variables (i.e. path analysis), a particular strength of SEM is in the modeling of unobserved latent variables (or *factors*), which are not measured in the datasets, but can be deduced based on the data. For example, tumor immune infiltration, a very important phenomenon in cancers, is not reported in the data, but can be deduced based on collective mRNA expression patterns of some key marker genes. Similarly, mitotic index of tumors, differentiation (or stemness) status, etc… are some of the examples that could be depicted as factors in SEM, and analyzed with relation to molecular and clinical data.

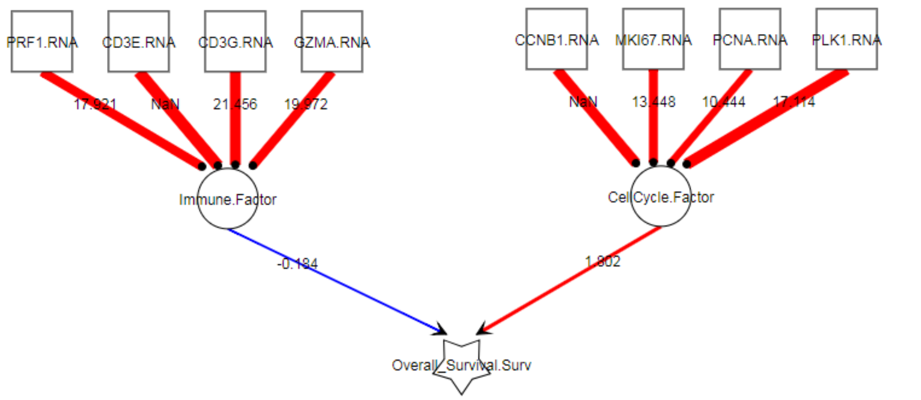

Figure 8. Results of factor analyses of the relative effect of immune cell infiltration and cell cycle progression on clinical outcome.

To demonstrate the use of latent factors, let's analyze how mitotic progression and cytolytic immune cell infiltration in tumors affects clinical outcome. Refresh the web page to load a new session of SEMA. Add following mRNA expression nodes to the graph: CCNB1, MKI67, PCNA, PLK1, PRF1, CD3E, CD3G and GZMA. Now, select "Factor" in the node type menu, and type "CellCycle", click "Add", and now type "Immune", click "Add". Now, select "Factor" from the "Link type" drop-down menu on the top-left, and draw "factor" links from CCNB1.RNA to the CellCycle.Factor node; you will notice that the factor link has a circle head. Draw factor links to the CellCycle.Factor links from MKI67.RNA, PCNA.RNA and PLK1.RNA nodes as well. We have now defined the CellCycle.Factor node. Define the Immune.Factor node same way by drawing factor links from CD3E.RNA, CD3G.RNA,

Parameter	Full name	Interpretation
**MODEL**	Model numer	These appear in the order of evaluation by SEMA
**DF**	Degrees of freedom	DF of a structural model is equal to the difference between known (variances and covariances of observed variables) and free parameters
**CFI**	Comparative Fit Index	A fit index measuring the fit of the model to the data. CFI ranges from 0-1. Values closer to 1, ideally > 0.9, are considered a good fit
**TLI**	Tucker-Lewis Index	Another fit index very similar to CFI.
**AIC.SEM**	The Akaike Information Criterion for the structural part of the model (excluding the survival part)	This is another comparative measure of fit. This is most useful when comparing different models. Lower values indicate better fit.
**SRMR**	Standardized root mean square residual	An absolute measure of fit. Measures average difference between the observed and the model-predicted covariances. Values < 0.1 are considered acceptable
**NTOTAL**	Total number of variables	
**AIC.COX**	The AIC for the survival part of the model from Cox regression	

Table 3. SEM model fit measures provided in SEMA for each model and their explanation.

GZMA.RNA and PRF1.RNA nodes. Select “Clinical” from the node type menu, and add “Overall_Survival” node to the model. Draw links from the CellCycle.Factor and Immune.Factor nodes to the Overall_Survival node. Click “Evaluate”. Select “z-scores” from “Mark links by” menu. The result should be like in Figure 8.

The thick lines of the factor loadings (i.e. factor links) indicate good factor definitions. Goodness-of-fit of the factor definitions and of the model as a whole can be tested by clicking on “Stats” button, and displaying the fit measures from the SEM analysis. Several fit measures are provided in this table for each evaluated model. Table 3 summarizes these measures and their meanings. You will notice that the fit measures for this model are acceptable (i.e. TLI and CFI > 0.9, and SRMR < 0.1 for most datasets).

Select the CellCycle.Factor -> Overall_Survival link, and click on “Plot selection” to visualize the Kaplan-meier plot. Select “Z-scores” in the “Plot for the link:” menu on top of the plot area, and compare the z-scores of the effect of cell cycle factor on the clinical outcome across adult and pediatric cancers. You will see an overwhelming strong positive effect (positive z-scores here mean correlation with poor outcome, negative z-scores mean correlation with better outcome) across cancers. Do the same for Immune.Factor -> Overall_Survival link, and you will see the opposite in most tumors, with the exception of blood and brain tumors, as expected.

As an exercise, add MYC and CDKN2A copy number variations (GISTIC) to the model, and see how these affect the cell cycle and immune infiltration processes in cancers. Also, you can try experimenting with other factors, such as “epithelial mesenchymal transition” (EMT), which can be defined by its marker genes (e.g. VIM, FN1, CDH1, CDH2, TWIST, SNAI1).

This completes the introductory tutorial of the basic functionalities in SEMA. Refer to the videos for sample case studies illustrating more advanced analysis capabilities in SEMA.

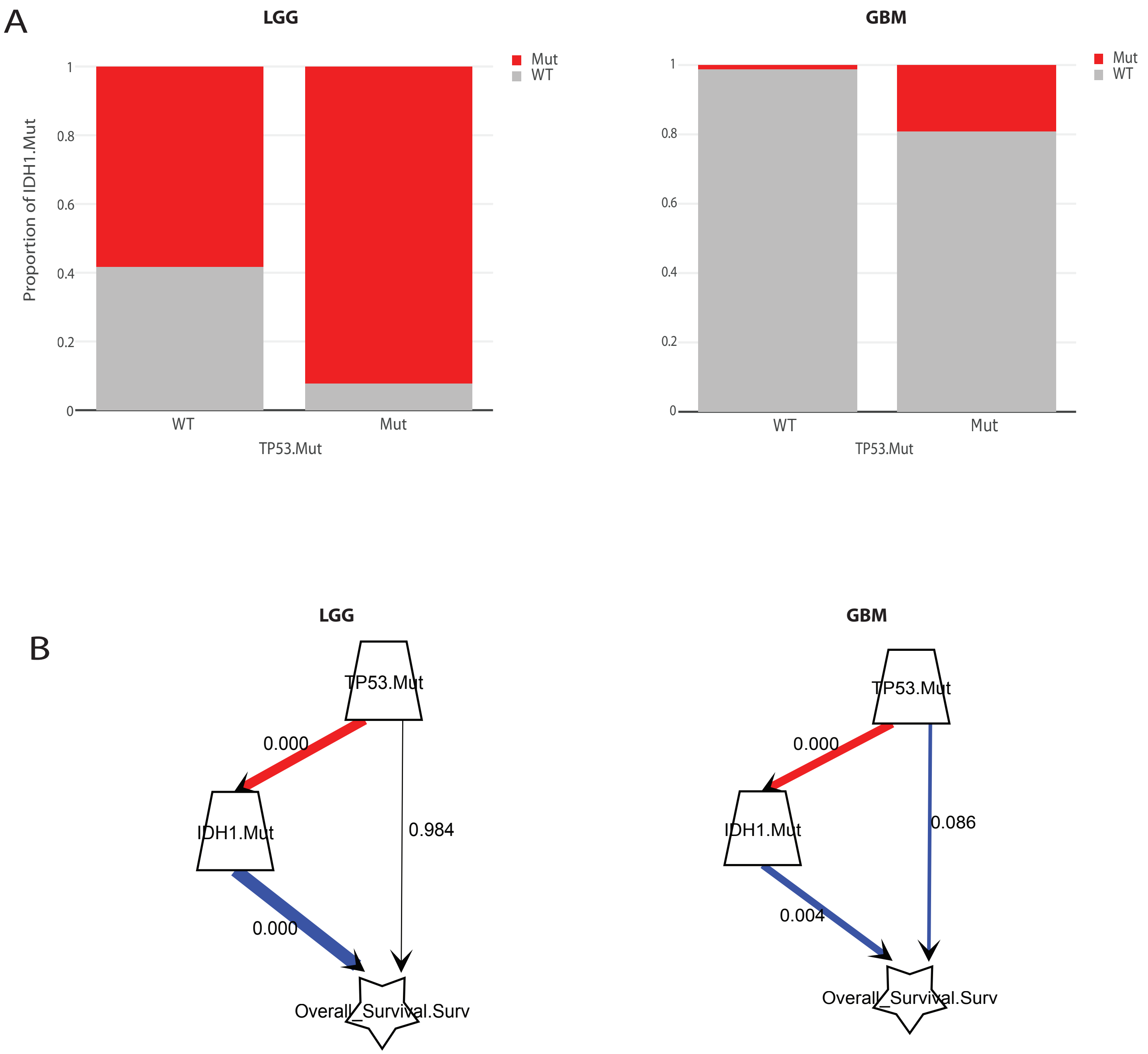
A
LGG
Mut
WT
Proportion of IDH1.Mut
1
0.8
0.6
0.4
0.2
0
WT
Mut
TP53.Mut
GBM
Mut
WT
1
0.8
0.6
0.4
0.2
0
WT
Mut
TP53.Mut
B
LGG
TP53.Mut
0.000
IDH1.Mut
0.984
0.000
Overall_Survival.Surv
GBM
TP53.Mut
0.000
IDH1.Mut
0.086
0.004
Overall_Survival.Surv

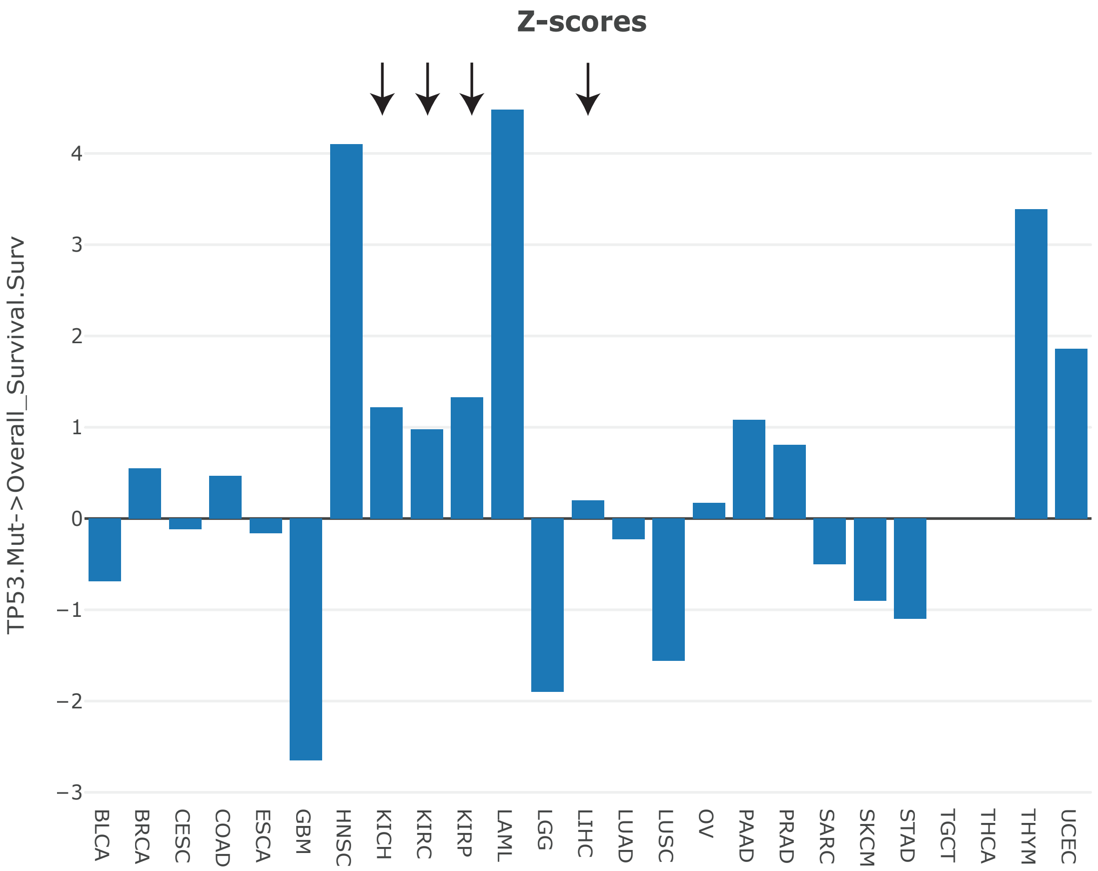

Z-scores
TP53.Mut->Overall_Survival.Surv
4
3
2
1
0
−1
−2
−3
BLCA
BRCA
CESC
COAD
ESCA
GBM
HNSC
KICH
KIRC
KIRP
LAML
LGG
LIHC
LUAD
LUSC
OV
PAAD
PRAD
SARC
SKCM
STAD
TGCT
THCA
THYM
UCEC

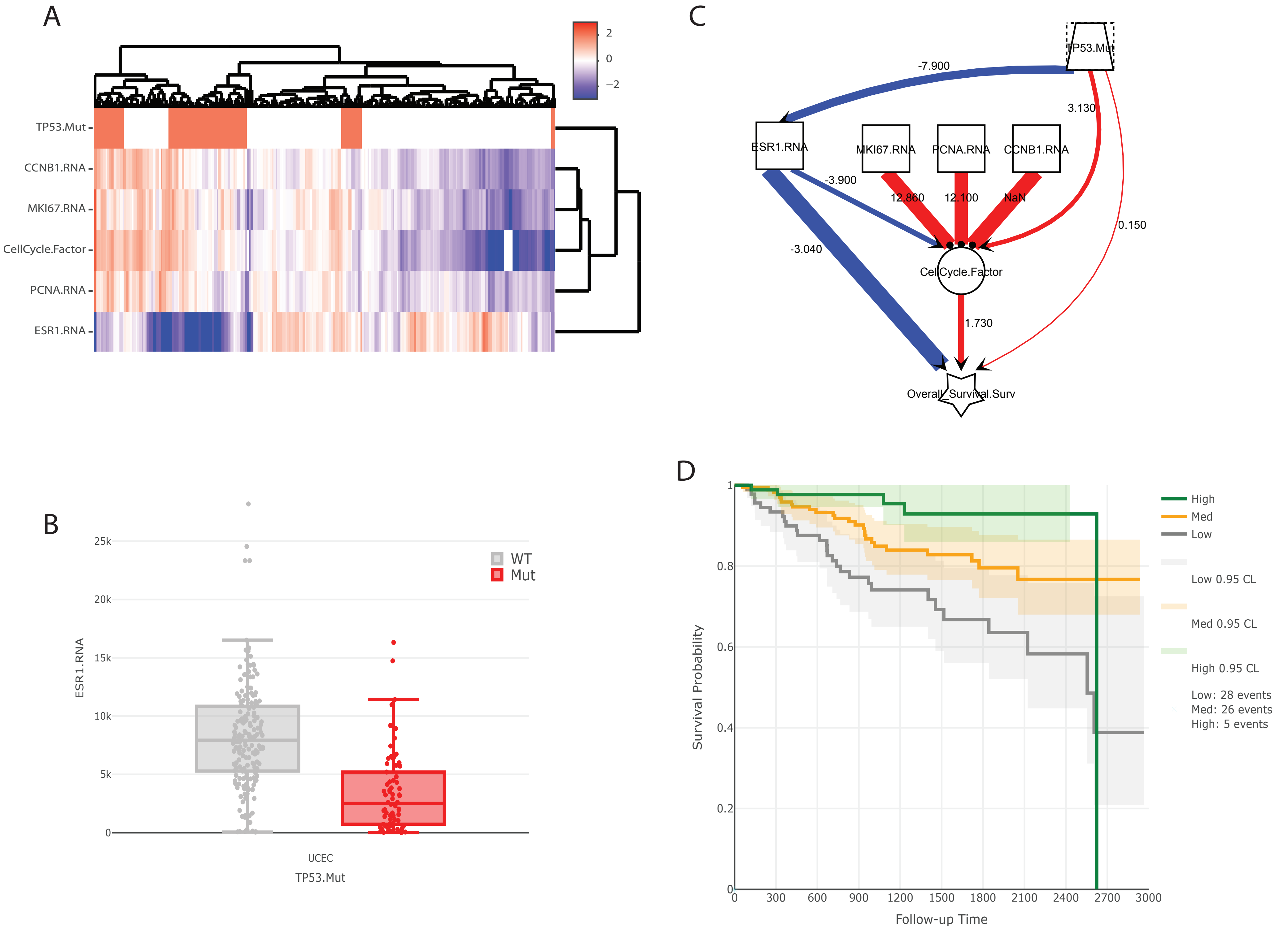
A
2
0
−2
TP53.Mut
CCNB1.RNA
MKI67.RNA
CellCycle.Factor
PCNA.RNA
ESR1.RNA
B
ESR1.RNA
25k
20k
15k
10k
5k
0
WT
Mut
UCEC
TP53.Mut
C
TP53.Mut
-7.900
3.130
ESR1.RNA
MKI67.RNA
PCNA.RNA
CCNB1.RNA
-3.900
12.860
12.100
NaN
0.150
-3.040
CellCycle.Factor
1.730
Overall_Survival.Surv
D
Survival Probability
1
0.8
0.6
0.4
0.2
0
0
300
600
900
1200
1500
1800
2100
2400
2700
3000
Follow-up Time
High
Med
Low
Low 0.95 CL
Med 0.95 CL
High 0.95 CL
Low: 28 events
Med: 26 events
High: 5 events
